# Optimizing sialorrhea management in Parkinson’s disease: a patient journey mapping approach

**DOI:** 10.3389/fneur.2026.1768568

**Published:** 2026-03-05

**Authors:** Huimin Guan, Xiaolin Ma, Chuyue Jiang, Hui Liu, Yuling Xu, Guangjian Zhang, Heng Liu

**Affiliations:** 1 School of Nursing, Qingdao University, Qingdao, Shandong, China; 2 Department of Traumatology, Qingdao Municipal Hospital, Qingdao, Shandong, China; 3 Department of Pain, Affiliated Hospital of Yanbian University, Yanji, Jilin, China

**Keywords:** nursing, Parkinson’s disease, patient journey map, qualitative research, sialorrhea

## Abstract

**Background:**

A troubling non-motor symptom of Parkinson’s disease (PD), sialorrhea greatly lowers quality of life and raises the risk of aspiration. But today’s clinical care is frequently disjointed. Although patient trip mapping provides a comprehensive method for identifying care gaps, its use in sialorrhea nursing strategy optimization is still somewhat unexplored.

**Objective:**

The purpose of this study is to use a thorough patient journey mapping methodology to clarify the unique difficulties and unfulfilled requirements that PD patients with sialorrhea have during their clinical management route. The resulting insights will be essential for directing the development of customized therapeutic interventions, guaranteeing the best possible patient-centered care.

**Methods:**

The design used was descriptive qualitative. Ten PD patients with sialorrhea who were recruited through local Parkinson’s disease patient associations or community networks in Qingdao City (July–September 2025) participated in semi-structured interviews. The data was subjected to thematic content analysis in order to outline the needs and experiences of the participants along the sialorrhea management pathway. The results were used to create a thorough patient path map.

**Results:**

Thematic analysis yielded 32 themes to construct a patient journey map (PJM)—a patient-centered visualization of care stages, actions, and emotional trajectories. The journey spans four stages: pre-consultation, consultation, treatment, and long-term management. Key findings include: (1) significant knowledge gaps pre-consultation; (2) communication hindered by complex jargon; (3) emotional ambivalence (hope vs. guilt) during treatment; and (4) adherence challenges due to stigma. This PJM effectively highlights critical pain points to guide service improvements.

**Conclusion:**

To sum up, the created patient journey map provides clinical personnel with an easy-to-use visual platform. The timely identification and strategic design of focused therapies are made possible by its effective delineation of patients’ sialorrhea management requirements and crucial pain locations across various phases. For people with PD and sialorrhea, this tool is crucial to maximizing the delivery of patient-centered care.

## 1 Introduction

Parkinson’s disease (PD) is the second most common neurodegenerative condition in the world, after Alzheimer’s disease, an estimated 1.37% of people in China who are 60 years of age or older have the condition (1), indicating a significant and expanding public health concern. PD is becoming acknowledged as a complex multi-system condition, while being historically characterized by core motor characteristics, such as bradykinesia, stiffness, and tremor (2). It includes a wide range of non-motor symptoms, including autonomic dysfunction, cognitive decline, sleep disorders, and neuropsychiatric symptoms like despair and anxiety (3). Between 32 and 74% of people with Parkinson’s disease experience sialorrhea, a common but often untreated autonomic symptom (4). Pathophysiologically, it is caused by intraoral saliva buildup and overflow, which is mostly caused by oropharyngeal dysphagia and a decrease in the frequency of spontaneous swallowing rather than hypersalivation (5). Persistent sialorrhea presents serious clinical hazards in addition to impairing oral hygiene, such as local infections, perioral dermatological issues, and possibly fatal aspiration pneumonia (6). As a result, chronic illness significantly reduces health-related quality of life, intensifies social disengagement, and places a significant financial strain on caregivers and healthcare systems (7).

Further in-depth research into the clinical pathway is necessary due to the significant impact of sialorrhea on the health-related quality of life of PD patients and the requirement for a truly comprehensive therapeutic approach. Pharmacological medicines, targeted injectable therapy, and, less frequently, deep brain stimulation (DBS) are the main treatment techniques used today. However, poor tolerance and unwanted anticholinergic side effects sometimes limit pharmaceutical alternatives, especially in the elderly population (8). Similar to this, there are still a lot of clinical questions about the best dosage and long-term therapeutic effectiveness of injections of botulinum toxin (9). Additionally, DBS has inherent surgical risks (such as bleeding or infection) and stimulation-related adverse events (such as the worsening of dysarthria or dysphagia, paresthesia, and mood or behavioral changes) (10), which could seriously compromise the patient’s overall improvements in quality of life.

Due to the shortcomings of traditional symptom management, the emphasis must change from treating the illness to comprehending the patient. Thus, it is crucial to correctly and thoroughly investigate patients’ holistic, multifaceted experiences and unmet demands during the whole course of their illness. The full range of patient-healthcare system interactions is graphically depicted by the patient journey map (PJM), a potent patient-centered tool. Its use is becoming more and more essential to the management of chronic diseases since it makes it possible to precisely see and identify important gaps in care and particular pain spots (11–14). The PJM frequently produces favorable results in a variety of specialized fields, providing significant practical utility within healthcare services. It directs intelligent and customized rehabilitation routes toward service improvement by visualizing the complete experience and multifaceted demands of patients, such as young and middle-aged stroke patients with dysphagia (15). The PJM provides strategic guidance to maximize clinical services, interdisciplinary teamwork, and home care assistance for older diabetic foot ulcer patients by progressively highlighting their requirements and pain points (16). Furthermore, examining tasks, emotions, and pain sites offers patients with chronic heart failure (CHF) specific support measures to maximize volume control and improve self-management abilities (17). Extending this utility, the PJM allows healthcare professionals to more comprehensively identify the core challenges and psychological needs of PD sialorrhea patients at different stages of their care, subsequently allowing for the precise optimization of service processes at each critical touchpoint.

Consequently, our study uses the PJM approach to methodically outline the comprehensive therapy continuum for PD patients suffering from sialorrhea, from the beginning of symptoms to long-term maintenance. The main goal is to accurately map the essential requirements and contextual obstacles that arise throughout the management process. In the end, the resulting data will provide a solid basis for improving patient-centered treatment and customizing and refining therapeutic intervention techniques.

## 2 Methods

### 2.1 Participants and setting

A convenience sample of 10 PD patients with documented sialorrhea was recruited through local Parkinson’s disease patient associations or community networks in Qingdao City. Recruitment took place between July and September 2025.

#### 2.1.1 Inclusion criteria

Participants were enrolled if they met all the following criteria:

(1) Diagnosis of PD confirmed according to the 2016 Chinese Diagnostic Criteria for PD.
(2) Presence of sialorrhea indicated by a score of ≥1 on Item 6 (Salivation) of the Unified PD Rating Scale, Part II (UPDRS-II).
(3) Provided informed consent and voluntarily agreed to participate in the study.

#### 2.1.2 Exclusion criteria

Patients were excluded based on the following:

(1) Diagnosis of secondary parkinsonism or Parkinson-plus syndromes.
(2) Presence of severe cognitive impairment, active psychiatric disorders, or severe visual/hearing deficits that would impede communication during the interview.
(3) Refusal to allow the audio-recording of the interview session.

#### 2.1.3 Sample size determination and ethics

Based on the principle of data saturation, a total of 10 PD patients with sialorrhea were ultimately included in the study. Their general demographic and clinical characteristics are summarized in Table 1.

**Table 1:** Demographic and clinical characteristics of PD patients with sialorrhea (*N* = 10).

Participant ID	Gender	Age	Occupational status	Place of residence	Living arrangement	Monthly income	Medical payment method	H&Y stage	Sialorrhea severity (UPDRS-II Item 6 score)
M1	Male	72	Retired	City	Spouse	6,000	Employee medical insurance	3	2
M2	Female	68	Retired	County town	Children	3,000	Resident medical insurance	4	3
M3	Male	60	Retired	City	Spouse	5,000	Employee medical insurance	2	1
M4	Male	78	Retired	Town	Spouse	2,000	Resident medical insurance	4	4
M5	Female	65	Retired	Village	Spouse	2,000	Self-pay	2.5	2
M6	Male	55	Employed	County town	Spouse	4,000	Employee medical insurance	1	1
M7	Male	70	Retired	City	Children	7,000	Employee medical insurance	3	3
M8	Female	81	Retired	County town	Hired caregiver	5,000	Resident medical insurance	5	4
M9	Female	58	Self-employed farmer	County town	Spouse	4,000	Employee medical insurance	2	1
M10	Male	63	Retired	Village	Spouse	2,000	Resident medical insurance	1.5	2

PD, Parkinson’s disease; UPDRS, Unified Parkinson’s Disease Rating Scale.

Ethical approval for this study was obtained from the Ethics Committee of Qingdao University (Approval No. QDU-HEC-2024407). Furthermore, the study was prospectively registered with the Chinese Clinical Trial Registry (ChiCTR), registration number ChiCTR2500096034 (available at: https://www.chictr.org.cn/index.html). Prior to participation, all participants provided written informed consent and were ensured that their involvement was voluntary.

### 2.2 Study design and procedures

This investigation was executed using a descriptive qualitative research design, which is particularly suited for in-depth exploration of human experiences. Primary data were collected through semi-structured interviews. The resultant findings were subsequently utilized to construct the PJM, strictly adhering to established PJM mapping methodologies (18).

#### 2.2.1 Development and validation of the interview guide

To ensure the relevance and methodological rigor of the data collection, a preliminary semi-structured interview guide was systematically developed. This process involved three critical steps: a comprehensive literature review, formal expert consultation (incorporating clinical and methodological expertise), and internal group discussions among the research team.

Subsequently, pilot interviews were conducted with four eligible PD patients (data excluded from the final analysis). The purpose of the pilot was to validate the clarity, comprehensiveness, and contextual relevance of the phrasing and content. Based on the feedback obtained, the research team iteratively refined and finalized the guide to ensure a thorough exploration of the authentic experiences, challenges, and unmet needs of PD patients across their sialorrhea management trajectory. The core interview domains and sample questions included:

(1) Initial symptom onset and awareness: Can you describe the first time you noticed the symptoms of sialorrhea? What were your immediate emotional reactions, and how did you initially attempt to manage it?
(2) Access to care and early consultations: During your initial medical consultation for sialorrhea, what specific barriers or difficulties did you encounter within the healthcare system?
(3) Diagnostic and therapeutic process challenges: What challenges or obstacles did you face during the diagnosis and subsequent treatment selection for sialorrhea?
(4) Critical touchpoints and decision-making: Throughout your overall treatment trajectory, which stages or decisions were the most pivotal or emotionally significant for you?
(5) Current self-management and efficacy: How do you currently manage your sialorrhea on a daily basis? What specific, adopted methods (pharmacological, non-pharmacological) have you used, and how do you evaluate their effectiveness?
(6) Socio-contextual support and influence: What roles have your family members, caregivers, or support network played throughout your experience with this condition? How has their involvement specifically influenced your management?
(7) Unmet needs and future expectations: Considering your journey, what specific needs remain unaddressed regarding sialorrhea management? What concrete suggestions do you have for improving clinical care or support services?

#### 2.2.2 Data collection

Participants’ contact information was initially retrieved via the hospital’s outpatient appointment system. Prior to each interview, the lead researcher provided a detailed explanation of the study’s purpose and procedures, ensuring participants understood their voluntary involvement. Written informed consent and confidentiality agreements were secured from every participant.

Each patient participated in a single semi-structured interview, lasting approximately 30 to 40 min, conducted in a private and quiet demonstration room to facilitate open discussion. All interviews were audio-recorded with the explicit permission of the participants.

To maximize the authenticity, accuracy, and comprehensiveness of the qualitative data, interviewers employed active interviewing techniques such as probing, rephrasing, and clarifying in real-time to meticulously verify participants’ perspectives and subjective experiences. Furthermore, the questioning style and sequence were flexibly adjusted, guided by the organic flow of the conversation. Data collection was formally terminated when data saturation was achieved.

#### 2.2.3 Data analysis and trustworthiness

To ensure analytical rigor, all audio recordings were transcribed verbatim and de-identified by two researchers within 24 to 48 h of each interview.

Conventional content analysis was applied to the textual data to systematically extract core concepts. Independent coding was first conducted by two researchers, followed by a collaborative review and consensus process to establish common experiences and ensure inter-coder reliability. The resulting codes were then iteratively categorized according to the distinct stages of the patient journey and the key elements of sialorrhea management until thematic saturation was established.

Finally, the emerging themes and the preliminary journey map were presented to the participants for member checking (validation) to refine the map’s structure and verify the completeness and accuracy of the information presented.

## 3 Results

### 3.1 Construction of the patient journey map

Following a comprehensive analysis and systematic integration of the qualitative interview data, the patient journey for sialorrhea management was delineated across four distinct, sequential phases: pre-consultation, consultation, treatment, and long-term management.

Across these four phases, the management experience and requirements of PD patients with sialorrhea were systematically synthesized into three critical dimensions: behaviors (actions), emotional responses, and critical pain points.

**Figure 1:**
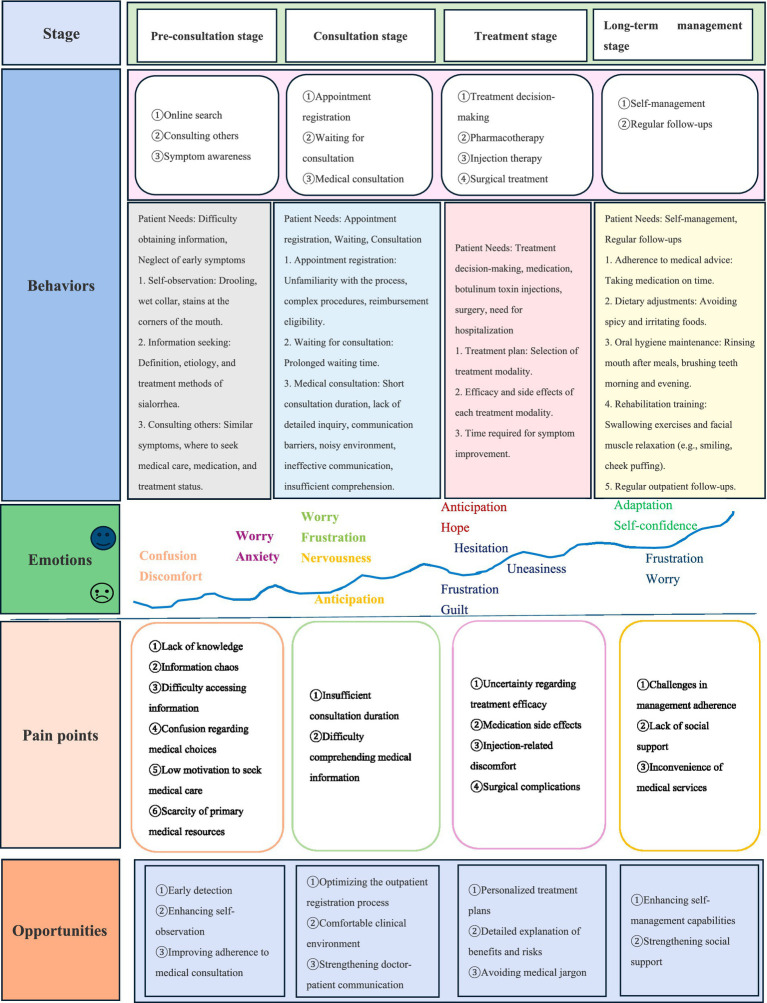
Comprehensive PJM of sialorrhea management in PD.

This structured synthesis culminated in the construction of a symptom management PJM for PD patients with sialorrhea, which visually represents the entire care pathway and serves as the conceptual framework for our findings (see Figure 1).

### 3.2 Pre-consultation phase

The pre-consultation phase was primarily characterized by patients’ initial efforts to understand and cope with the emerging symptoms of sialorrhea.

#### 3.2.1 Behaviors

##### 3.2.1.1 Difficulty in accessing reliable information

Participants attempted to seek information regarding sialorrhea through various channels, yet the information encountered was frequently chaotic and lacked verifiability. This struggle often led to initial misattribution of the symptom.

M6: “I searched for drooling online, and a lot of information stated it was an oral problem. I didn’t know PD could also cause drooling.”

M10: “I asked around among friends and relatives, but everyone gave different information, so I didn’t know who to trust.”

##### 3.2.1.2 Neglect and delay in addressing early symptoms

Due to insufficient attention paid to the initial, mild manifestations of sialorrhea, patients often missed the optimal opportunity for early intervention. This delay was driven by a lack of awareness of the symptom’s clinical significance.

M4: “At first, the corners of my mouth were just a little wet, so I didn’t take it seriously. Later, I realized it was getting worse and worse.”

#### 3.2.2 Emotions

##### 3.2.2.1 Confusion and discomfort

The initial emotional response was characterized by confusion and discomfort. Participants reported a profound lack of clarity regarding the etiology and optimal management of their sialorrhea, which significantly amplified their distress.

M1: “Drooling makes me feel very embarrassed. I have absolutely no idea what is going on or what I should do.”

##### 3.2.2.2 Worry and anxiety

A secondary, yet prevailing, theme was worry and anxiety. Patients expressed considerable concern that their symptoms would irrevocably damage their quality of life, specifically impacting self-image and inhibiting social engagement.

#### 3.2.3 Pain points

The analysis revealed five critical pain points that impede patients’ ability to effectively manage PD-related sialorrhea and seek appropriate care.

##### 3.2.3.1 Deficits in disease-specific knowledge

Patients reported a limited understanding of PD-related sialorrhea, which made it difficult to accurately assess their own condition and distinguish their symptoms from those of other comorbidities. This knowledge gap often led to misinterpretation and distress.

M2: “This drooling is really embarrassing. People think I have dementia, but the doctor says I don’t. I just don’t know what is actually going on with this saliva.”

##### 3.2.3.2 Information overload and verification difficulties

Participants struggled with the dilemma of information quality and volume. The sheer quantity of unregulated information available online was overwhelming, making it nearly impossible to differentiate between valid medical advice and unreliable sources.

M3: “There is too much information online about PD drooling. Some say take pills, others say get injections, plus all kinds of folk remedies. I don’t know which is true and which is fake.”

##### 3.2.3.3 Ambiguity in navigating medical choices

Faced with a multitude of therapeutic options (e.g., traditional Chinese medicine, Western pharmacotherapy, injections) and various specialty institutions, patients often experienced decision paralysis, rendering them unable to make informed choices about optimal treatment pathways or selection of a healthcare provider.

M7: “Facing all these methods—traditional Chinese medicine, Western medicine, injections, pills—and specialist clinics at several hospitals, I don’t know which one to choose or which hospital is the best place to go.”

##### 3.2.3.4 Barriers to timely healthcare utilization

A significant cluster of factors contributed to low motivation for seeking prompt medical care. These included logistical challenges (unfamiliarity with registration processes, transportation difficulties), a lack of local support (absence of nearby children or caregivers), and financial pressures, compounded by the tendency to underestimate the severity of sialorrhea.

M5: “I live in a village and can’t get to the county town on my own. Once you go to the hospital, they require all sorts of tests, which costs a lot. Drooling is a minor problem, so I felt there was no need to get it checked.”

##### 3.2.3.5 Scarcity of specialized primary care resources

Patients frequently encountered resource limitations within community-level health institutions. These primary care settings often lacked specialized professionals and sufficient medical capability to effectively diagnose and manage the specific symptom of PD-related drooling.

M9: “Our community hospital doesn’t even have a doctor who specializes in PD drooling. I went last time, and the doctor said he didn’t really understand it either; he just prescribed some medicine casually, which didn’t help at all.”

### 3.3 Consultation stage

#### 3.3.1 Behaviors

The consultation stage was characterized by two core areas of difficulty: challenges related to navigating the medical system and communication barriers during the patient-physician encounter.

##### 3.3.1.1 Information needs regarding medical access and logistics

Patients demonstrated unfamiliarity with the procedural logistics of healthcare access, creating significant barriers even before the consultation began. These difficulties included a lack of knowledge regarding appointment registration processes, managing waiting times, and understanding complex administrative issues like medical insurance reimbursement.

M10: “The appointment registration process is too complicated; it took me a long time to figure it out.”

M9: “While waiting for the consultation, I waited for a long time but didn’t dare go to the toilet because I was afraid of missing my turn.”

M10: “My insurance is paid collectively by the village, and I don’t know if it can be reimbursed if I go to a big city.”

##### 3.3.1.2 Communication deficits and information asymmetry

The second challenge involved substantial communication barriers during the clinical encounter. Participants reported that brief doctor-patient consultation times prevented them from fully articulating their condition. This temporal constraint, combined with the use of technical medical jargon, resulted in information asymmetry—patients were left with an insufficient understanding of their diagnosis and the rationale behind the proposed treatment plans.

M2: “The doctor used a lot of technical terms that I didn’t really understand. I still don’t know which is more effective: medication or surgery.”

#### 3.3.2 Emotions

The consultation stage triggered a sequence of complex emotional responses, shifting from initial anxiety and hope to eventual frustration and acceptance.

##### 3.3.2.1 Nervousness and anticipatory hope

Patients initially experienced a combination of nervousness and anticipatory hope throughout the diagnostic process. The apprehension stemming from the uncertainty of the diagnosis was counterbalanced by a strong expectation of finally finding an effective solution or a clinician capable of providing relief.

M9: “I was both nervous and full of anticipation, hoping to meet a good doctor who could cure the disease.”

##### 3.3.2.2 Worry and frustration driven by diagnostic confirmation

Upon the confirmation that sialorrhea was an integral manifestation of their underlying PD, the emotional state transitioned to one of worry and frustration. This shift was rooted in the dispelling of their original, often less severe, expectations and the forced confrontation with the reality of a chronic condition.

M6: “I originally thought it was a minor issue that would get better in a few days; I never expected it was PD.”

#### 3.3.3 Pain points

##### 3.3.3.1 Perceived insufficiency of consultation duration

Patients consistently reported insufficient consultation duration as a major source of dissatisfaction. The brevity of the interaction, especially following the difficulty of securing an appointment, led to a fragmented communication process where patients felt their narrative was incomplete and many of their critical questions remained unaddressed.

M4: “The consultation time was too short. It was so hard to get an appointment, yet before I could even explain a few things clearly, the doctor was already sending me for tests or prescribing medication directly. I didn’t have time to ask many of my questions.”

##### 3.3.3.2 Challenges in comprehending medical information

A significant information asymmetry was identified, stemming from patients’ difficulty comprehending the medical information presented by physicians. The use of technical terminology (jargon) to explain disease pathology and proposed treatment plans indicated a fundamental need for more detailed, simplified, and accessible explanations to ensure patients understood the actual benefits, risks, and rationale behind their care.

M1: “The doctor used a whole bunch of technical terms with me, like ‘anticholinergic drugs’ and ‘botulinum toxin injections. ‘I couldn’t understand them at all, and I couldn’t figure out the actual benefits and risks of these treatments.”

### 3.4 Treatment stage

#### 3.4.1 Behaviors

##### 3.4.1.1 Navigating the treatment decision-making dilemma

The initiation of treatment began with a critical decision-making dilemma. Patients required comprehensive communication and guidance from their physicians to effectively weigh the options and finalize the most appropriate treatment modality, reflecting their need for clarity in the prescribed care pathway.

M1: “The doctor explained different treatment plans to me, but which one should I choose?”

##### 3.4.1.2 Initiation of pharmacotherapy or botulinum toxin injections

Following the decision phase, patients initiated either pharmacological treatment or received botulinum toxin (BoNT) injections to manage their sialorrhea. This step represented the commitment to a less invasive, targeted approach, with patients expressing both compliance and hope for symptom relief.

M3: “I take the medicine every day exactly as prescribed, hoping it will be effective.”

M6: “I hesitated for a long time but finally decided to get botulinum toxin injections, hoping for a speedy recovery.”

##### 3.4.1.3 Election of surgical intervention

In cases where non-surgical methods were deemed insufficient or as a primary recommendation based on clinical assessment, patients elected to undergo surgical intervention. This choice, often accompanied by some apprehension, was driven by the goal of achieving a more definitive or effective long-term control of sialorrhea and associated symptoms.

M4: “The doctor recommended surgery. Although I am a bit worried, I decided to go ahead with it to improve the drooling and other symptoms.”

#### 3.4.2 Emotions

The treatment phase elicited a complex emotional spectrum, encompassing ambivalence regarding the intervention, optimistic hope, and negative feelings rooted in unmet expectations and financial burdens.

##### 3.4.2.1 Ambivalence: hesitation and uneasiness

Patients reported significant hesitation and uneasiness primarily centered on treatment safety and efficacy. This anxiety manifested as apprehension regarding the potential for adverse drug reactions (ADRs) and the uncertainty surrounding the long-term outcomes of pharmacotherapy or injections.

M7: “I worry about whether the medicine has side effects. Recently, I’ve had a dry mouth and nausea, and I don’t know if it’s caused by taking the pills.”

##### 3.4.2.2 Motivation: anticipation and hope

Despite their fears, patients maintained strong anticipation and hope, driven by the positive expectation that adhering strictly to the prescribed regimen would lead to effective control and mitigation of their sialorrhea symptoms.

M7: “I am undergoing treatment exactly as the doctor requested, so there should be some improvement, right?”

##### 3.4.2.3 Disappointment and frustration

A distinct feeling of frustration arose when patients felt their concerns were minimized or when immediate, satisfactory therapeutic solutions were unattainable. This was often triggered by a perceived lack of clinical empathy or a failure by physicians to fully acknowledge the debilitating social and physical impact of the condition.

M8: “As soon as I open my mouth, there is a lot of saliva. With so many people in the room, I was too embarrassed to talk much, and the saliva also makes my speech unclear. Yet, the doctor was too busy to ask me about it in depth.”

##### 3.4.2.4 Financial strain and guilt

Finally, patients expressed deep feelings of guilt related to the substantial economic burden of ongoing treatment. They often perceived the considerable financial outlay as an undue imposition, positioning themselves as a burden on their family members and caregivers.

M8: “Spending money on this disease is like pouring water down the drain; it costs several thousand every month. I feel like I’m a burden on my children, but I can’t just leave the disease untreated.”

#### 3.4.3 Pain points

The post-treatment phase presents a distinct set of pain points related to efficacy, adverse events, and surgical outcomes.

##### 3.4.3.1 Uncertainty regarding treatment efficacy and duration

Patients consistently expressed uncertainty regarding treatment efficacy and the sustainability of therapeutic benefits. This ambiguity fueled anxiety about the required duration of pharmacological commitment and the timeline for observing measurable symptom improvement.

M8: “I don’t know how long I have to take this medicine before it becomes effective.”

##### 3.4.3.2 Adverse events from medication or injection-related discomfort

A common pain point involved direct adverse events, including reported ADRs and post-injection discomfort. Patients often found it difficult to attribute specific symptoms (e.g., dizziness, pain) to the intervention, increasing their overall distress.

M4: “That medicine the doctor prescribed… after taking it, I felt very dizzy, so much so that I couldn’t even stand steadily.”

M7: “Recently, the spot where I got the botulinum toxin injection has been aching vaguely, and I have a bit of a headache too. I don’t know if it’s a side effect of the injection or some other reason.”

##### 3.4.3.3 Concerns over surgical complications

For those undergoing operative procedures, a primary concern was the potential for surgical complications. Patients articulated fears of risks such as infection, nerve injury, and the disheartening prospect of suboptimal postoperative outcomes, which would invalidate the physical and emotional burden of the procedure.

M6: “Although the doctor said the surgery results are quite good, I heard that someone got an infection after having it done. If it turns out to be ineffective, I will have suffered for nothing.”

### 3.5 Long-term management stage

#### 3.5.1 Behaviors

The long-term management of sialorrhea was characterized by patients’ active engagement in self-care and strict adherence to the medical protocol.

##### 3.5.1.1 Proactive self-management and non-pharmacological interventions

Patients actively implemented proactive self-management strategies, focusing on non-pharmacological interventions and lifestyle adjustments to mitigate symptoms. These activities included rigorous oral hygiene maintenance, engaging in targeted swallowing retraining, and conscious postural adjustments.

M2: “I rinse my mouth more often now, practice swallowing movements, and try my best not to eat spicy food.”

##### 3.5.1.2 High treatment adherence and systematic monitoring

Participants demonstrated high levels of treatment adherence by consistently following prescribed rehabilitation protocols and medication schedules. Furthermore, they committed to attending regular follow-up visits, which were necessary for the systematic monitoring and evaluation of symptom improvement and the overall efficacy of the treatment regimen.

M3: “I follow the doctor’s instructions by taking my medicine every day and coming in for regular follow-ups. I also do rehabilitation exercises on my own every day.”

#### 3.5.2 Emotions

The long-term management phase was characterized by a prevailing sense of adaptation and self-efficacy, tempered by intermittent psychological vulnerability related to the risk of relapse.

##### 3.5.2.1 Adaptation and enhanced self-efficacy

As effective symptom control was achieved, patients demonstrated successful adaptation to their chronic condition, leading to an increase in self-efficacy and the regain of self-confidence. This emotional shift positively impacted their willingness to re-engage with social activities.

M7: “The drooling is much better now, so I have the courage to go out.”

##### 3.5.2.2 Vulnerability: occasional frustration and anxiety

Despite overall improvement, the emotional state remained vulnerable. Occasional frustration and anxiety persisted, often triggered by minor relapses of sialorrhea or instances of public social embarrassment. The core anxiety centered on the fear of recurrence or the instability of their current controlled state.

M10: “I feel the drooling isn’t as severe as before, but I just occasionally worry about whether it will relapse.”

#### 3.5.3 Pain points

The enduring nature of chronic disease management introduced three key pain points that undermined sustained well-being and consistent healthcare access.

##### 3.5.3.1 Challenges in sustaining management adherence

Patients reported significant challenges in sustaining adherence to the required long-term management regimen. The cognitive and physical burden of consistently maintaining exercises and protocols over an extended period often led to fatigue and a struggle with compliance.

M10: “Sticking to these exercises every day… sometimes I really do feel tired.”

##### 3.5.3.2 Deficits in social understanding and support

A critical issue was the lack of social support and understanding from the broader community. The stigma associated with visible symptoms often resulted in negative social interactions and judgment, significantly impacting patients’ psychological well-being and sense of comfort in public.

M9: “Sometimes people around me give me strange looks, which makes me feel very uncomfortable.”

##### 3.5.3.3 Systemic inconvenience of medical services and access barriers

Patients continued to face systemic inconvenience in accessing medical services. This was primarily driven by geographical barriers, which necessitated long travel distances and inconvenient transportation to specialized hospitals, especially as local, community-level institutions were deemed medically inadequate for their specific needs, resulting in a suboptimal healthcare experience.

M10: “I live in a village. I have to go for a follow-up every two weeks, and the round trip takes several hours. The township hospital here isn’t good enough, so I have no choice but to go to the big city.”

## 4 Discussion

### 4.1 Optimizing primary care services to promote timely medical consultation

Early detection, fast diagnosis, and appropriate medication initiation are essential for the successful management of sialorrhea in patients with PD during the pre-consultation stage. However, the results of this study show that major delays in consultation are caused by a combination of poor motivation to seek medical care, substantial obstacles to information acquisition, and inadequate disease knowledge. The symptoms of sialorrhea are undoubtedly made worse by this postponement, which in turn causes negative affective states as worry, shame, and low self-esteem (19). Therefore, in order to greatly improve patients’ capacity to recognize symptoms and their perception of the need for professional medical assistance, healthcare teams must actively participate in public health education regarding the prevention and management of PD-related sialorrhea.

It is specifically advised to take a multifaceted approach.

Prioritizing the development of primary care staff’s professional capability is the first step. To improve their ability to correctly identify and treat PD-related sialorrhea, this training should concentrate on early detection, differential diagnosis, and early intervention techniques (20). Second, innovative health education approaches should be used by basic healthcare institutions. To spread important information about the causes and consequences of PD-related sialorrhea and to highlight the importance of early consultation, it is crucial to use a variety of formats, such as focused community lectures and easily accessible online popularization platforms (such as official WeChat accounts and brief video content). This tactic aims to reduce the social stigma connected with the illness and help patients and their families have a proper knowledge of it.

Additionally, individualized health counseling services must to be set up to directly respond to patient questions and clear up common misunderstandings. Patients will have easier access to trustworthy data and professional resources if information technology is strategically used to build authoritative information-sharing platforms. In the end, these combined strategies will support sialorrhea early identification, precise diagnosis, and prompt treatment in PD patients at the foundational level.

### 4.2 Optimizing clinical workflows and strengthening patient-physician communication

Improving patient-physician communication and streamlining clinical procedures are essential for raising the standard of medical care and greatly improving the patient experience during the consultation phase. According to this survey, lengthy wait times, complicated appointment registration procedures, and ineffective patient-physician communication are major issues that need to be resolved right now. Medical facilities should apply optimization strategies in the following two main areas to successfully solve these systemic issues.

First, reducing operational stages must be the top priority for optimizing clinical workflows. In addition to the active introduction of technologies like online appointment scheduling, intelligent triage systems, and self-service terminals, the implementation of “one-stop” services that include registration, triage, examination, and payment (21) can significantly reduce patient waiting times and unnecessary navigation within the hospital setting. Additionally, giving patients easy-to-use medical guidelines and flowcharts would make it much easier for them to finish the consultation procedure.

Second, improving patient-physician communication requires medical personnel to continuously take an active, patient, and diligent attitude during the phases of diagnosis and treatment. In particular, this entails attending to the patient’s main concerns in full during the consultation; giving thorough, understandable explanations of the condition, suggested treatment plans, prognosis, and possible risks; and patiently answering all questions from patients and their families. Medical professionals should avoid using too much specialist medical jargon and instead use plain, everyday English to facilitate optimal comprehension. Simultaneously, setting aside specific times for in-depth patient-physician communication and using digital platforms for additional health education and follow-up can promote a more cordial and trustworthy working relationship, which will ultimately improve patient treatment adherence and satisfaction.

### 4.3 Developing personalized treatment plans to achieve precise patient intervention

In patients with PD, sialorrhea is exceedingly heterogeneous, with complicated pathogenic pathways and multiple factors leading to a wide range of clinical manifestations (22). The results of this study demonstrate that when patients are presented with a variety of therapeutic alternatives, including medication, botulinum toxin (BoNT) injections, or surgical intervention, this significant individual variability frequently causes decision-making challenges. To achieve exact intervention, it is therefore essential to create a patient-centered, customized treatment strategy.

In order to precisely identify the underlying etiology, this precision requires a thorough and complete first evaluation that includes measuring the severity of sialorrhea, assessing swallowing function, and examining salivary gland secretion. Building on this objective diagnosis, the patient’s overall illness status, comorbidities, current drug schedule, cognitive state, and particular quality-of-life needs must all be fully taken into account. In order to ensure the patient’s right to informed consent and active participation in the decision-making process, treatment goals should be jointly determined based on patient preferences and followed by a thorough disclosure of any potential negative effects.

Treatment regimens should use a phased and varied approach based on this extensive examination and communication. Anticholinergic drugs, lifestyle changes, and specialized oral rehabilitation advice should be the main strategy for people with mild to moderate sialorrhea. Ultrasound-guided botulinum toxin injections provide a feasible route for targeted management in the event that pharmaceutical efficacy proves inadequate or serious side effects manifest. Only after a thorough risk–benefit analysis may surgical treatment be considered for the small number of patients whose illness is resistant to all other treatments, whose quality of life is seriously impaired, and who meet strict surgical indications.

In order to ensure that regimens are modified in real-time depending on observed efficacy and side effects, the entire treatment process requires dynamic monitoring and feedback. PD patients with sialorrhea can achieve precise management and notable symptom improvement thanks to this iterative approach, which ensures that interventions stay closely linked with changing patient demands.

### 4.4 Enhancing patient self-management and strengthening social support

Long-term self-management is an essential extension of patient care since sialorrhea is a chronic, progressive condition for which there is presently no dramatic solution and because symptoms tend to worsen as the disease progresses (23). Our study found that inadequate social support and poor adherence to the treatment plan are important variables for the disease to worsen and quality of life to decline. Establishing a multifaceted, integrated intervention approach that aims to significantly improve the self-management skills of patients with PD-related sialorrhea and boost their access to social support is therefore both essential and crucial.

Personalized information empowerment and training in practical skills are crucial for self-management (24). Patients should be helped to gain a thorough understanding of the pathophysiology of sialorrhea through comprehensive disease education. At the same time, students need to learn a number of useful management skills, such as accurate medicine administration, focused oral motor exercises, and efficient swallowing methods. Patients should be routinely encouraged to keep a sialorrhea diary and make full use of digital health tools (such mobile applications) to help with medication reminders and guided rehabilitation in order to promote self-monitoring and accurate assessment. These strategies, which are complemented by positive behavioral incentives, are intended to progressively foster proactive and successful self-management behaviors, assisting patients in gaining self-assurance in facing the illness and actively managing everyday obstacles.

In terms of social support, building a strong foundation of support requires a multifaceted strategy. This entails enhancing family caregivers’ professional training to make sure they have both the technical caregiving skills and the ability to offer effective psychological assistance. At the same time, strong patient support networks that smoothly combine online and physical channels should be aggressively established. These peer-support groups greatly lessen patients’ feelings of loneliness and social shame by facilitating the sharing of personal experiences and emotional solace. Additionally, integrating community medical and rehabilitation resources, improving primary healthcare staff’s professional skills, and expanding public health awareness campaigns are all crucial to improving patients’ quality of life holistically and creating a more compassionate and encouraging long-term rehabilitation environment.

### 4.5 Limitations

First, regarding the sample and geographical scope, the small sample size (*N* = 10) and single-site recruitment from a specific city (Qingdao, China) may limit the transferability of the results. As noted, healthcare systems, insurance policies, and cultural attitudes toward PD vary significantly across regions and nations; thus, the identified patient journey and pain points may lack external applicability in different international contexts. Therefore, future multi-center studies involving diverse geographical regions are warranted to validate and refine these findings.

Second, the representativeness of the patient population is constrained by two critical factors. On one hand, to ensure reliable qualitative communication, patients with severe cognitive impairment were excluded. This means the study may underrepresent the experiences of more advanced PD populations who often face the most severe sialorrhea and the most complex management challenges. On the other hand, this study focused exclusively on PD-related sialorrhea. As sialorrhea management is highly dependent on the underlying etiology, the PJM and associated pain points for PD patients might differ significantly from those experiencing sialorrhea due to other factors, such as clozapine therapy. Therefore, future research utilizing caregiver-proxy reports and comparative etiological analyses is warranted to provide a more holistic and cross-diagnostic understanding of sialorrhea management.

Third, a key dimension missing from this research is the caregiver’s perspective. Since many PD patients are highly reliant on their caregivers—particularly for medication administration, oral hygiene maintenance, and daily symptom monitoring—the caregivers’ priorities and the specific aspects of care they find most challenging are integral to the management pathway. While this study prioritized the patient’s own lived experience to establish a baseline journey, future research should incorporate caregiver insights to provide a more holistic view of the sialorrhea management ecosystem.

Finally, the study relied on retrospective self-reports, which may introduce recall bias, potentially affecting the precision of the emotional and behavioral data captured from earlier stages of the patient journey.

## 5 Conclusion

Our study methodically outlines the primary behaviors, feelings, and pain areas of patients with PD-related sialorrhea over the whole care continuum by creating a patient journey map. This visualization method improves patient self-awareness, encourages efficient patient-physician communication and treatment compliance, and gives medical practitioners an empirical foundation for dynamically optimizing resource allocation and service workflows.

## Data Availability

The original contributions presented in the study are included in the article/supplementary material, further inquiries can be directed to the corresponding author.
